# Rheology of Highly Filled Polymer Compositions—Limits of Filling, Structure, and Transport Phenomena

**DOI:** 10.3390/polym16030442

**Published:** 2024-02-05

**Authors:** Alexander Ya. Malkin, Valery G. Kulichikhin, Svetlana Yu. Khashirova, Igor D. Simonov-Emelyanov, Anton V. Mityukov

**Affiliations:** 1 A.V. Topchiev Institute of Petrochemical Synthesis, Russian Academy of Science, 29. Leninsky Prospect, 119991 Moscow, Russia; klch@ips.ac.ru (V.G.K.); ant-mityukov@yandex.ru (A.V.M.); 2 Kh.M. Berbekov Kabardino-Balkarsky State University, Chernyshevsky Str. 273, 36000 Nal’chik, Russia; new_kompozit@mail.ru; 3 M.V. Lomonosov Institute of Fine Chemical Technology, Russian Technological University, 78. Vernadsky Avenue, 119454 Moscow, Russia; igor.simonov1412@gmail.com

**Keywords:** polymers, solid particles, dense suspensions, jamming, heterogeneity

## Abstract

The current state of the rheology of various polymeric and other materials containing a high concentration of spherical solid filler is considered. The physics of the critical points on the concentration scale are discussed in detail. These points determine the features of the rheological behavior of the highly filled materials corresponding to transitions from a liquid to a yielding medium, elastic–plastic state, and finally to an elastic solid-like state of suspensions. Theoretical and experimental data are summarized, showing the limits of the most dense packing of solid particles, which is of key importance for applications and obtaining high-quality products. The results of model and fine structural studies of physical phenomena that occur when approaching the point of filling the volume, including the occurrence of instabilities, are considered. The occurrence of heterogeneity in the form of individual clusters is also described. These heterogeneous objects begin to move as a whole that leads to the appearance of discontinuities in the suspension volume or wall slip. Understanding these phenomena is a key for particle technology and multiphase processing.

## 1. Introduction

Polymers are almost always used in a wide variety of compositions, including filling a large volume of solid components. In many cases, it is desirable to use high concentrations of filler. Typical examples of objects of this kind are solid rocket propellants, armor protection formed by dilatant (thixotropic) compositions, aircraft coatings, etc. Another class of highly filled polymer materials includes the compositions used for powder injection molding and additive technology. In such compositions, the content of a solid component can reach up to 75 vol. %, and the polymer can play the role of both a binder and the main functional material. The rheological properties of all these objects are important in many major manufacturing operations related to polymer processing.

A fantastic variety of highly filled polymeric materials, from the point of view of physical chemistry, can be characterized using the general term suspensions. Such a universal view on powders dispersed in the fluid matrix allows us to approach the assessment of their structure and properties from the same general position. In this respect, the rheological properties of such dispersions, which are directly related to the structure of the suspensions, are of basic interest. Varying the concentration, size, and shape of the particles, as well as the interaction of the dispersed particles with each other and with a continuous fluid phase, allows materials with a variety of desirable properties to be obtained. All these factors also affect the rheological properties of the suspensions and, thus, play a decisive role at the stage of chemical engineering to obtain final products.

Naturally, a complex of problems associated with the properties and structure of suspensions has been and remains the subject of study not only of technologists but also of research in the field of physics, colloid chemistry, and rheology, the purpose of which is to understand the fundamental aspects of the structure of suspensions and the relationship of the structure with their rheological properties.

In this regard, we have prepared the present review, which considers the current state of the field of suspension rheology, with particular attention to the latest publications. It is practically impossible to review, in detail, all aspects of the area under consideration in one publication. The problems associated with yielding liquids (transition from solid-like state to flow) in multicomponent systems have been recently considered in reviews [1,2]. Fundamental ideas about the peculiarities of the rheological properties of complex media related to the problems discussed in this review have become the subject of a number of recent systematic discussions [3,4,5].

Therefore, here, we considered it necessary to discuss such central issues of highly loaded materials including the evolution of the structure of suspensions and its dependence on the concentration of solid particles via an analysis of the physical nature of the concentration critical points. Next, we will consider the achievement of the maximum possible concentration, which is fundamentally important for obtaining high-quality products in some technologies including powder blow molding. At the same time, the physics and rheological behavior of the suspension when approaching the critical degree of filling will be considered in detail and, finally, the formation of heterogeneous structures with transition to macroscopic displacement will be described.

## 2. Structure as a Function of the Concentration

When solid particles are introduced into a continuum medium, there are two characteristic concentrations determining the structure of the dispersion.

First, this is the concentration at which a three-dimensional structure arises that exists throughout the entire volume of the substance. This is the percolation threshold, φ*. The structure formed in the volume has a greater or lesser strength. Therefore, at concentrations of the dispersed phase, the suspension is in a solid (or gel-like, if the particles of the dispersed phase are relatively easily deformed) state and at the concentration φ* the yield stress $\sigma_{Y}$ appears.

The yield stress characterizes the strength of the structure formed by a disperse phase and, in this sense, is a measure of cohesive forces. Meanwhile, in case of colloidal gels, many other forces like attractive ones (van der Waals and electrical/magnetic forces) can all lead to a yield stress [6]; it is possible to estimate the lower possible threshold of the yield strength, based on the fact that it must exceed the strength of the structure destroyed by Brownian motion. As shown in [2], this value is common for any soft matter and is determined using the Péclet Number:

(1) $$Pe=\frac{\sigma_{Y}d^{3}}{kT}>>1$$

where *d* is the characteristic size of the structural element, *T* is the absolute temperature, and *k* = 1.380649·10^−23^ J/K is the Boltzmann constant.

This implies a possible yield limit:

(2) $$\sigma_{Y}>>\frac{kT}{d^{3}}.$$

For the characteristic size of the particles that form the structure, it is reasonable to take the typical (arbitrary assumed for optically scattering colloidal particles) size of colloidal particles or the wavelength of visible light (0.4 µm). Hence, it follows that the minimum possible strength of the structure in yielding media can be no lower than 0.01 Pa, which roughly corresponds to the minimum values of the yield strength obtained by extrapolating the experimental data.

Currently, we are far away from a historical understanding of the yield strength described in the pioneering work of Bingham. A modern understanding of the concept of yielding was discussed in several recent publications [7,8,9]. 

Shear stress, σ, is an external factor breaking this structure. Then, it is reasonable to assume that the ratio $\sigma /\sigma_{Y}$ is responsible for intensifying the dismemberment of the structure formed during the disperse phase. This approach was approved in the qualitative analysis of dismembering droplets in concentrated emulsions [10] and effectively applied in examining structural changes under shearing [11]. In this publication, the ratio $\sigma /\sigma_{Y}$ has been presented using the Mason Number, *Mn*, which is considered as the ratio of the external mechanical force to the cohesive force and is expressed as

(3) $$Mn=C6\pi \varphi_{eff}^{2}\frac{\sigma }{\sigma_{Y}}$$

where *C* is an empirical constant and $\varphi_{eff}$ is the effective concentration of the dispersed phase.

Using the unique experimental technique, the authors measured the structural elements of the length scales spanning from nanometers to tens of micrometers and found that the characteristic size of the structure elements is proportional to $Mn^{-1}$.

The value of the critical concentration for the formation of a percolation network φ* can be estimated using the Monte Carlo method for the accepted model of a structural network forming a “continuous cluster” [12,13]. In the most general form, it was presented by Dean [14]. The model is based on the adopted coordination number Z, which determines the number of contacts of a single dispersion particle with the surrounding particles. Using the minimum possible Z = 2, φ* = 0.15–0.17. However, larger values of Z are more realistic and then φ* increases. So, for Z = 3, 4, and 5, the theory gives, respectively, φ* = 0.255, 0.34, and 0.43. It has been experimentally shown that a solid-like structure, characterized by the existence of a yield point, appears in suspensions at a concentration of the order of φ* = 0.281 ± 0.003 [15] and, taking into account the interlayer formed between the critical particles, φ* = 0.34 [16].

Indeed, the formation of a solid-like structure can occur at much lower concentrations of dispersed particles if their size goes into the submicron region.

In decreasing the size of particles, the tendency to agglomeration becomes a more and more critical factor. This is especially important for nanoparticles. Therefore, the percolation threshold is determined not so much by the concentration as by the formation and destruction of agglomerates of nanoparticles. In addition, shear-induced breakdown and agglomeration is of a kinetic nature, and the liquid-solid transition in yielding media becomes dependent on the history of deformations, which shifts the concentration threshold [17].

Considering the threshold of percolation as the cause of the yielding transition can be wrong and does not take into account the possibility of specific interactions between dispersed particles. When considering the microstructure that determines the rheological properties of dispersions, specific (attractive or repulsive) interactions between particles can play a certain role. However, in hard-sphere suspensions hydrodynamic interactions are dominated by lubrication interactions, while in soft colloidal suspensions, lubrication interactions are reduced significantly due to the repulsive force, and the colloidal forces are, thus, dominant in setting the structure [18].

The yield point in supramolecular structures can arise at mean surface to volume concentrations of the order of 0.5% [19] or even 0.06% [20]. Obviously, this is due to the sharply increasing surface of dispersed particles as their size decreases.

Interparticle interaction can be discussed in the terms of friction [21], which definitely shifts the φ*. Meanwhile, there are a lot of other chemical factors related to the quality of the particle surface and the nature of the continuous phase, which strongly influence the conditions of the structure formation and the value of the yield stress. However, consideration of this area of physical and colloidal chemistry is far beyond the scope of this review.

The second characteristic parameter, determined using the structure of concentrated suspension, is the threshold of maximal possible filling of the volume using solid non-deformed (spherical) particles *φ_m_*, and above this threshold there is no empty space for adding even a single particle. Of course, the particle packing structure depends on the particle interaction (attractive or repulsive), which ultimately should affect the rheological properties of the material. The role of particle interaction is an independent big issue that requires separate consideration. In this review, we will not touch on it, considering the particles as neutral, discussing the phenomena and effects at a qualitative level. In this case, the influence of particle interaction will be reflected only in the fact that, in quantitative estimates, it will lead to a certain range of the values under discussion.

In this case, the influence of particle interaction will be reflected only in the fact that, in quantitative estimates, it will lead to an uncertain change in the values under discussion. Meanwhile, it is reasonable to assume that interparticle interaction becomes influential if the gap between particles is less by order than the size of the particles. Then, the value of this factor is determined by the potential function describing the interaction and this function may be very different. Moreover, in this case, is necessary to distinguish whether these particles are in an ordered state (and then it is possible to place more particles in the volume) or whether they fill the volume in a statistical (random) disorder. Depending on the nature (geometry) of the package, the maximum degree of filling varies somewhat, and the maximum value is equal to $\pi \sqrt{2}/6≅0.74$, achieved via hexagonal packing of balls of any size. With statistical packing, the effect of repulsion of particles due to steric interactions is possible; in their absence, the extremely large degree of packing is often determined by geometric reasons due to the lack of free volume. It is the latter case that is of general importance for determining the limiting value of random packing *φ_m_*. For monodisperse spherical particles this limit lies in the range of 0.5–0.6. Sequential computer simulation of three-dimensional dense packing of spherical particles was performed and gave the limiting value of *φ_m_* for a bidisperse mixture depending on the ratio between large and small particles; f_m_ this value varies between 0.64 and 0.78 [22].

Direct measurement of *φ_m_* has been made via free filling with balls of various sizes of a fixed volume according to bulk density. As it turned out, for sufficiently large particles *φ_m_* = 0.488 ± 0.004 (starch particles of 14 µm in diameter) and 0.576 ± 0.004 (glass spheres of 88–125 µm in diameter) [6].

However, a decrease in the particle size reduces the value of *φ_m_*, which can decrease for nanoparticles to 0.05. This is due to the formation of strong but loose clusters that do not collapse even at very high compression pressures of nanoparticles [23]. Therefore, it is impossible to introduce a significant amount of solid filler consisting of nanoparticles into the matrix. Typical values of *φ_m_* depending on the particle size of the filler are given below.

**Figure 1:**
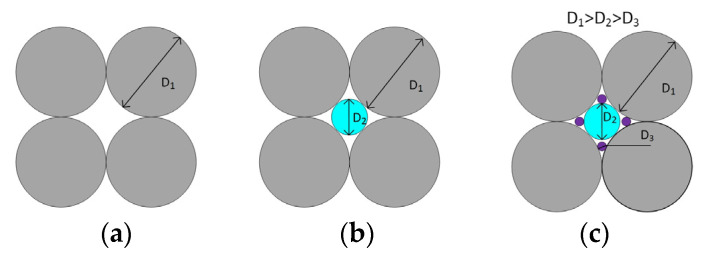
Filling the volume by controlling the particle size distribution of the bidispersed phase: mono-(**a**), bi-(**b**), and ternary (**c**) distributions.

The main way to increase the content of solid particles above the limits indicated in Table 1 is to switch from monodisperse particles to polydisperse ones by adjusting their particle size distribution. This approach is illustrated in Figure 1, which shows how smaller particles fill the voids formed between larger particles. The general theory of volume filling using polydisperse particles was considered in a number of works (e.g., [24]). It was noted that the limiting degree of filling to some extent depends on the viscosity of the medium in which the solid particles are placed [25]. The solution of the specific problem of increasing the limiting degree of volume filling is based on the following rather obvious considerations.

The optimal ratio of particle sizes in a bidispersed mixture (as in Figure 1b) is found from the ratio of the diameters of larger *D*_1_ and smaller *D*_2_ spherical particles.

From Figure 1 it follows that the optimal ratio of particle sizes in a bidispersed mixture is determined using the following formula:

(4) $$D_{2}=D_{1}\left(\frac{1}{\cos30^{{}^{\circ}}}-1\right)≅0.157$$

So, the particle diameter of the finer fraction *D*_2_ should be ~6.5 times smaller than the diameter *D*_1_ of the main coarser fraction. (The size ratio in Figure 1b is shown conditionally, since it does not correspond to this value). Then the volume that can be occupied by a finer fraction is 0.231, and the total maximum possible content of the filler reaches—*φ_m_* = 0.64 + 0.231 = 0.871.

The maximal filling appears dependent on the size ratio of powder particles and the relative volume fraction of the large ones. Of course, in all cases the limiting concentration in the random packing is less than the limit for regular packing *φ_m_*. The rheological properties of bidispersed suspensions, as well as the possible filling limit, and the conditions of jamming depend on the nature of the interaction between the particles. Appropriate results, taking into account short-range lubrication forces, frictional interaction, and repulsion between particles, are presented and discussed by Malbranche et al. [26]. The rheology of bidispersed, non-Brownian suspensions using particle-based simulation, mapping the viscosity as a function of the size ratio of the species, their relative abundance, and the overall solid content, has also been studied by Singh et al. [27].

The continuation of similar calculations for the third fraction shows that, in a three-component system, the maximum possible particle content *φ_m_* reaches 0.928. The introduction of a fourth even finer fraction into the system increases *φ_m_* only up to 0.938, i.e., it is a practically negligible effect on the possibility of increasing the limiting concentration of the filler.

Actually, the achieved concentrations of a disperse phase are even less than predicted using a model of random packing. In practice, the approach to model estimations can be reached via the forced migration of particles in mixtures, and this can be realized via vibrations creating the effect of apparent “melting”, which increases the mobility of solid particles in suspensions [28].

Thus, there are two characteristic concentrations of solid particles in the liquid matrix that correspond to the formation of a percolation structure and yield strength φ* and the concentration at which the limit of the dense packing of solid particles in the volume *φ_m_* is achieved. These concentrations correspond to changes in the rheological state of the suspension. So, at φ < φ* the suspension is a viscous liquid, and in the range of concentrations between φ* and *φ_m_* this is a *yielding medium*.

**Figure 2:**
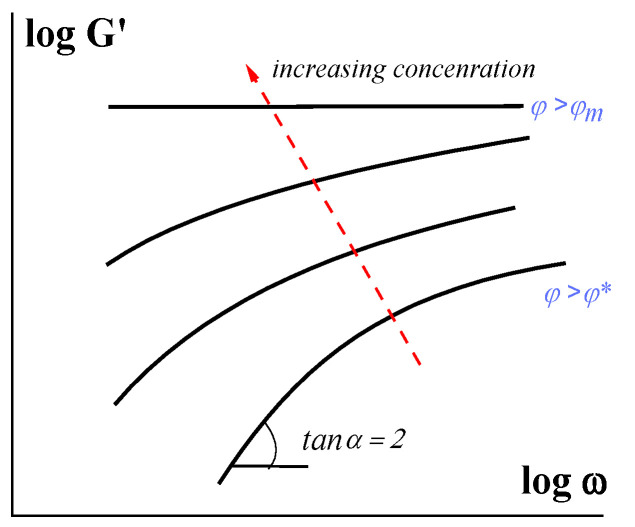
Evolution of the elastic (storage) modulus in increasing the concentration of a solid phase.

The transition to the concentration region *φ* > φ* is characterized by the other non-linear effects, besides yielding. There are non-Newtonian flow curves and linear and non-linear viscoelastic phenomena [29,30]. With an increase in concentration, the Maxwellian type of viscoelasticity is continuously changed to a decrease in the exponent in the frequency dependence of the elastic (storage) modulus $G^{\prime }∼\omega^{n}$ from 2 to zero [31,32]. The last situation (*n* = 0) corresponds to a solid-like state of dense suspensions. This typical situation is shown in Figure 2.

The non-linearity of viscoelastic properties in periodic oscillations manifests itself in a decrease of the elastic modulus while increasing the strain amplitude, and the limit of linearity is usually observed at very small amplitudes of strains since the structure formed by solid particles is brittle. However, at least in some cases, such an unusual effect is observed as the preservation of the harmonic form of stress changes in the nonlinear region during the set of sinusoidal strains [33].

The estimate of the concentration φ* is unambiguous only for a monodisperse suspension. The rheological properties and achievement of the yielding state of polydisperse suspensions, of course, are highly dependent on the composition of the particles, since they are controlled by frictional and adhesive contacts [34].

Meanwhile, two sub-regions should be distinguished within these two regions, in which the rheological properties of the suspension are significantly different. So, in the region *φ* < φ*, one can distinguish a range of very low concentrations, in which the dependence of viscosity on concentration is linear, and regions of higher concentrations, at which, when representing the dependence, it turns out to be necessary to take into account nonlinear effects (for any analytical description of this dependence). This limiting concentration of linearity can be estimated based, for example, on the well-known expression in the form of a quadratic function. Different coefficients at the quadratic term have been proposed, and a typical value is close to 5.0 [35].

(5) $$\eta /\eta_{0}=1+2.5\varphi +5\varphi^{2}$$

It follows that the quadratic term can be neglected approximately at *φ* < 0.02, so we can assume that *φ_l_* ≈ 0.02; in the region *φ* > *φ_l_*, some nonlinear effects such as non-Newtonian flow can be observed.

**Figure 3:**
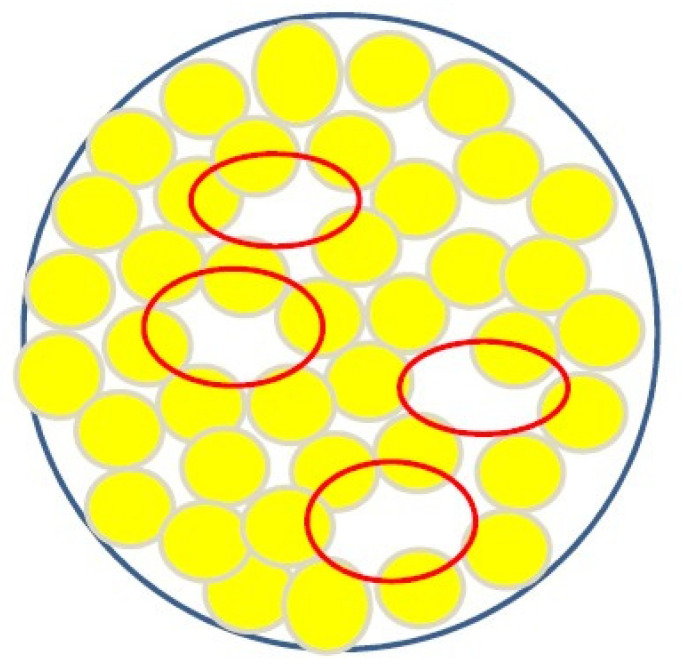
Empty spaces (shown as red ovals) when the volume is randomly filled using spherical particles.

One more characteristic concentration can be identified in the region close to *φ_m_*. The concentration range between φ* and *φ*_m_ is considered as the concentration range when the suspension flows at stresses exceeding the yield point. Then, under the action of constant stresses, irreversible deformations develop; only at concentrations close to *φ_m_* does the flow become impossible. However, due to the fact that with randomly created dense packing voids remain in the volume (as shown in Figure 3), particles can irreversibly shift inside these voids. Yet, this does not create a flow, since such movements are limited by the local volumes (shown by the ovals in Figure 3) and do not lead to a displacement in the entire volume. Therefore, plastic deformations (depending on stress) occur inside these rooms, but they are restricted and do not develop over time. Thus, a region of elastic–plastic behavior of a concentrated suspension arises close to *φ_m_* [31,36].

The concentration and details of the microstructure of concentrated suspensions are important for their macroscopic rheological properties. Correlations between microstructure and macroscopic properties have been demonstrated using the unique experimental technique of micro-rheo-mapping, based on multiparticle tracking experiments, that allowed researchers to obtain an accurate and direct visualization of the microstructure [37]. An analysis of the structures created by thickeners with different high degree of crosslinking, which promote aggregates of different size and space distribution, showed that the type of microstructure heterogeneity determines the bulk elasticity of dispersions.

## 3. Approaching the Limit of Filling

“Approaching to the limit of filling” means that we are dealing with so-called “*dense*” suspensions, in which the resistance to the relative displacement of particles is determined by intermolecular microscopic forces as well as by dry friction mechanics depending on the surface roughness of the contacting particles [38].

**Figure 4:**
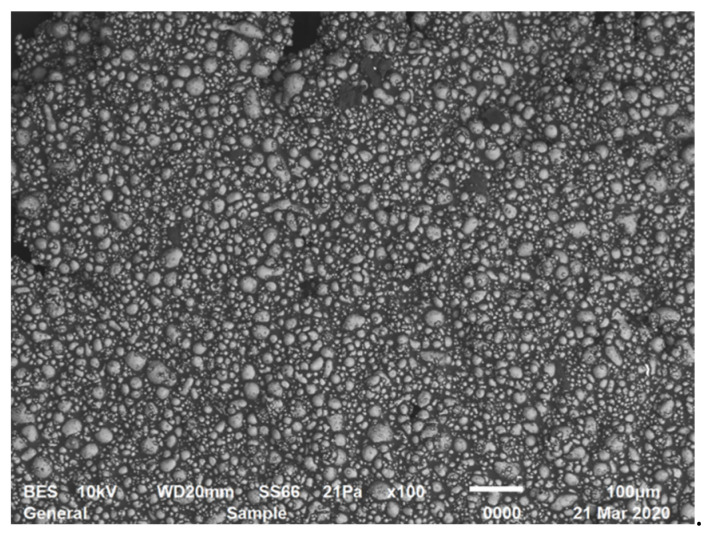
Microphotograph of a 60% suspension of aluminum powder (scanning electron microscope JSM-6510 LV, JEOL, Akishima, Japan).

The example of the suspensions with high concentrations close to the limit of filling is presented in Figure 4. The voids (back spots) between densely packed particles in a highly filled composition are clearly visible.

According to the old concept of Wo. Ostwald (1929), the dependence of non-Newtonian viscosity on the shear rate was called “structural viscosity” which was observed hundredfold as a decrease in apparent viscosity as a function of the shear rate and is related to the thixotropy of the fluid [39]. However, studies on the rheology of concentrated suspensions opened a different and richer picture associated with the structural effects. A comprehensive review of earlier studies devoted to shear-induced structural transformation was presented by [40].

**Figure 5:**
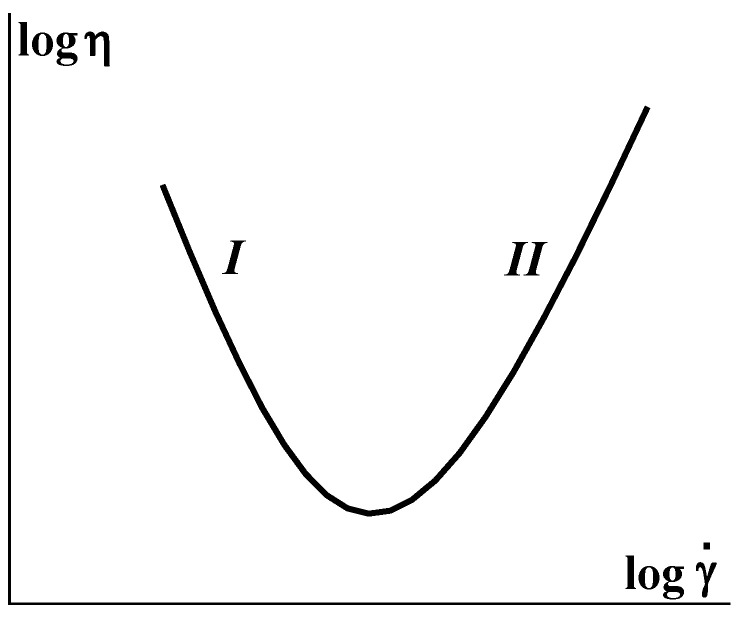
Dependence of the apparent viscosity on shear rate for concentrated suspensions of rigid particles.

An increase in the apparent viscosity along with an increase in the shear rate has been described for suspensions of rigid particles with concentrations of *φ_m_* close to 0.5–0.55 [41,42]. Figure 5 shows a decrease in apparent viscosity in the range of a low shear rate (***I*** in Figure 5), which is replaced by an increase in apparent viscosity at a high shear rate (***II*** in Figure 5). The branch ***II*** in this dependence is also considered as a dilatancy of the fluid. Dilatancy of concentrated suspensions was observed not only in shearing but also in extensional flows [41].

The formation/destruction of clusters has often been considered as a possible mechanism for shear thinning. Such clusters were observed in numerous experiments [43,44,45]. The formation of clusters was also observed in numerous works using optical and scattering methods, for example, the small-angle neutron scattering (SANS) method (e.g., [46]). In this case, there was a direct correction between the rheological properties and the detected structure.

The structure of the clusters can be completely disordered. However, the development of layering and planar order, and even (as an exceptional case) packing into hexagonal crystal layers (with numerous defects) between walls that slide past each other, was also observed [47].

These clusters create inhomogeneous structures in bulk, which are the source of plugs leading to jamming. The latter term is commonly used to describe the ceasing of flow at high shear rates. The consequence of deformation is also the occurrence of anisotropy, since the deviation from uniformity occurs in time and consequently leads to thixotropic/antithixotropic phenomena [48]. This phenomenon is associated with a shear-activated or deactivated network of contacts between particles [49].

Shear-induced ordering leading to anisotropy of the structure and anisotropic properties is especially characteristic for dispersed rods in extensional flows [50], although this phenomenon can be expected for shearing-dense suspensions.

Jamming continues to be a subject of permanent interest and numerous publications. Different manifestations of this effect exist. The dilatancy shown in Figure 5 is classified as *continuous shear thickening*—CST, while it can also happen in a jump-like manner at some critical shear rates which is classified as *discontinuous shear thickening*—DST.

**Figure 6:**
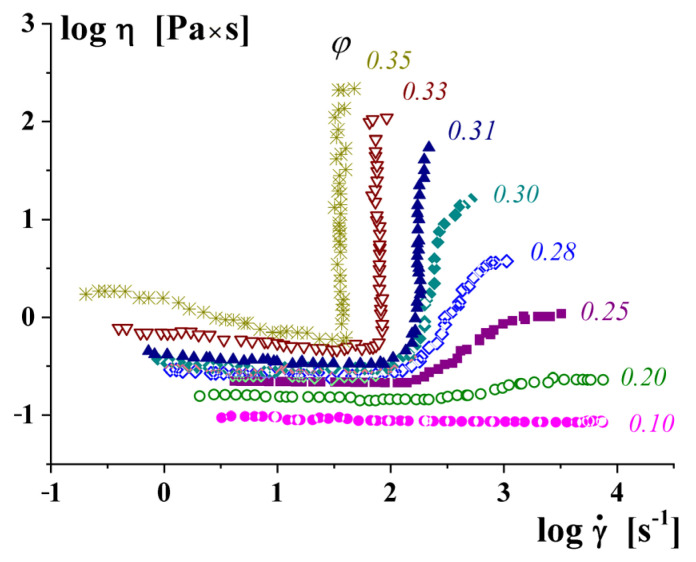
The transition from CST to DST when increasing the concentration of a disperse phase CaCO_3_ in poly(ethylene glycol) [51] (with permission).

The CST–DST transition takes place when increasing the concentration of a disperse phase, as seen from Figure 6.

**Figure 7:**
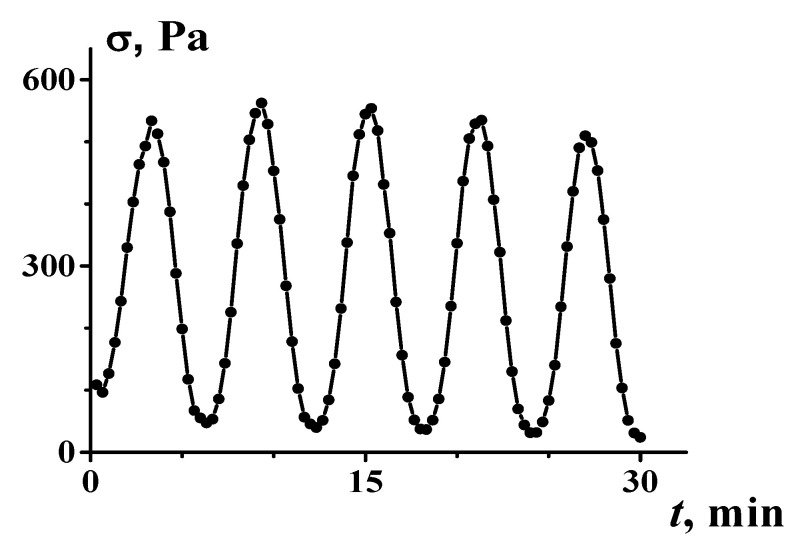
Self-oscillations in shearing the 56 mass. % suspension of *α*-FeOOH (Goethite) in transformer oil at a constant given global shear rate [53].

The transition to the solid-like state (which can be treated as an analogue of the mechanical glass transition) can be accompanied by such a phenomenon as “phase” separation. Of course, this is not a real thermodynamic effect, although an initially homogeneous suspension separates into two different domains with different concentrations (higher and lower). One of them is solid-like and the other can flow (see more details in the next section). This separation is unstable and leads to a rather interesting phenomenon of self-oscillation (at the given global shear rate) associated with the passing from one “phase” to the other or the kinetics of the breakup–creation of clusters. A rheological picture of the phenomenon is shown in Figure 7.

Usually characteristic effects, associated with structure formation in highly concentrated black carbon suspensions, were observed when the shear rate was varied (as in Figure 6). However, results similar in meaning can also be obtained via nonlinear oscillations in the regime of large deformation amplitudes (LAOS), as has been shown, for example, in [52].

The physics behind shear thickening is a subject of constant debate. The fundamental experimental evidence (for colloidal-size particles) is that this phenomenon is driven by the continuous transition from homogeneous colloidal flow to granular flow [54] that correlates with numerous observations of the cluster formation for non-colloidal particles. This result allowed for scaling the critical stress for the onset of dilatancy as *d*^−2^ (*d* is the particle diameter in suspensions).

A more general approach to the theory of shear thickening includes the consideration of hydrodynamic and contact (friction) mechanisms. Under increasing shear rates, particles become “locked together giving rise to large lubricant connected *hydrocluster*s that resist flow”, although it was found that contact forces play a dominant role [55].

Wang et al. [56] developed a detailed hydrodynamic model of DST. According to this model, the dominant contribution to the flow resistance is associated with hydrodynamic stresses between interacting colloidal particles and these stresses increase in the decrease in the separation gap between two particles. The theory shows that the spatial stress distribution at the onset of DST is highly localized. The visualization of the hydrodynamic frictional particle coupling is considered in [57]. The possibility of an inertial mechanism of shear thickening and the transition from a viscous to an inertial regime of jamming was considered in [58].

It is believed that jamming, determined by friction between solid particles and the threshold concentration, depends on the coefficient of friction varying between *φ* = 0.55 for very high friction and *φ* = 0.64 for frictionless contacts [59]. In addition, it is shown that the inertial forces cannot be responsible for jamming, and a reasonable approach to understanding the jamming at high concentrations should associate it with friction between solid particles in dense suspensions [60]. Simulation of the friction model and various predictions depending on the possible parameters of this model were discussed in [61]. One of the important factors influencing the increase in viscosity of highly concentrated suspensions is the roughness of the particles [62]. Roughness increases the packing fraction. For example, it was shown that hydrodynamics alone are fully sufficient for generating DST for rough particles [63]. The role of friction in shear thickening increases with increasing roughness of the particle surface and the jamming concentration decreases with increasing surface roughness [64].

The onset of DST looks like a kind of phase transition where the fluid losses mobility and transforms into a solid-like state. Meanwhile, the difference between these effects is associated with the scale effect, since crowding effects may induce a glass transition for Brownian particles or a jamming transition for non-Brownian systems [65]. This problem is also discussed by Singh [50] and Morris [66].

This transition is related to the friction forces connecting dispersed particles [67]. This phenomenon occurs at concentrations below that of the closest packing concentration, and, therefore, DST can be linked with hysteresis after the applied pressure is removed. This approach to understanding DST has been explored by relating shear jamming to the first normal stress difference *N*_1_: in the flow region *N*_1_ < 0; the DST criterion is met when *N*_1_ becomes positive [68]. The role of normal stresses in DST has been also examined by Prabhu and Singh [69].

Of primary interest are the physical events that occur in the closest approach to DST. Clusters appeared at lower concentrations. Model calculations showed that when approaching *φ_m_*, just below jamming, large scale instability, strong fluctuations, and bifurcation of the flow curve are observed, accompanied by strong oscillations of stresses and the formation of unstable stress bands [70]. This picture is similar to the one shown in Figure 6 but demonstrates a more chaotic nature.

CST has the same origin but arises as a consequence of averaging unsteady, spatially heterogeneous flows characteristic to DST. This model description of the phenomena arising in approaching the jamming concentration corresponds with visual observations of localized fluctuations and the formation of the band structure and a layer with limiting packing [71,72]. Instability, which manifests itself in stress fluctuations at the CST stage, can be suppressed and shear thickening weakens with an *increase* in the viscosity of the liquid continuous phase. These experimental results mean that viscous damping decreases apparent friction and reduces force correlation among particles [73]. Meanwhile, the incorporation of a small amount of liquid into dry powders via repulsive interactions smooths out the thickening effect and makes it possible to implement the flow regime [74].

When concentrated suspensions flow in channels, especially in channels with internal restrictions (such as built-in gratings), congestion may also occur due to the formation of plugs. This is the clogging effect, which is superficially similar to the jamming phenomenon discussed here, but it is a phenomenon completely different in its mechanism [75].

Dry powders are the extreme case of suspensions in which the continuous liquid phase has been removed. Meanwhile, such powders can move irreversibly, which looks like a flow; in this case, an analogy with concentrated suspensions suggests itself. In an early review [76], the movement of dry powders (granules) was considered, which could move via relative rotation and macroscopic responses in terms of shear strength determined via the relative input of rolling and sliding friction. Steady-state shear strength (an analogue of viscous resistance) was possible in two regimes controlled by either rolling resistance or sliding friction.

General understanding of the mechanism and manifestation of shear thickening, i.e., an increase in apparent viscosity with an increasing shear rate, was proposed in an excellent review by Morris [77]. Here, first of all, we should abandon the comparison found in the literature between shear thickening and dilatancy, if by the latter we understand Reynolds dilatancy. The mechanism of the latter is the tendency for sheared wet sand (suspension) to expand. However, it has nothing to do with the shear thickening mechanism. As for the shear thickening mechanism, as discussed above, various authors associated it with phenomena such as order–disorder, hydrocluster, and lubricated-to-frictional transition. Morris believes that the determining mechanism is lubricated to frictional contact interactions, although only this model somewhat simplifies the real scenario.

## 4. Heterogeneous Displacements

**Figure 8:**
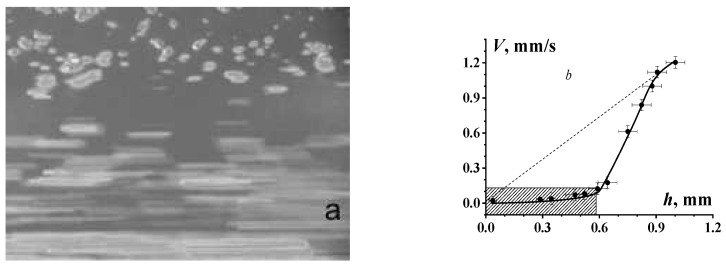
Photo of suspension flowing in the channel with a height of 1.0 mm (**a**) and the corresponding velocity profile (**b**). The shaded field is the area near the wall, the dotted line is the average flow velocity. The experiments have been performed using app. 40 vol. % suspension of *α*-FeOOH in water [78].

Considering rheological (as well as any others) properties implies that we are dealing with a homogeneous medium and the measured properties refer to any points in its volume or we have the right to average these properties over a chosen volume. This is correct for a wide range of concentrations but becomes unacceptable in dense suspensions due to the formation of clusters discussed in Section 3. These clusters begin to act as independent units and displacement occurs along their boundaries. The initial stage of this phenomenon is the appearance of an inhomogeneous concentration profile across the direction of flow. This is seen in Figure 8 where one can see the excess concentration of solid particles (served as markers) near the wall and the movement of separate particles in the central part of the flow. The liquid is moving in the horizontal direction due to the movement of the bounding surface (see details in [78]).

**Figure 9:**
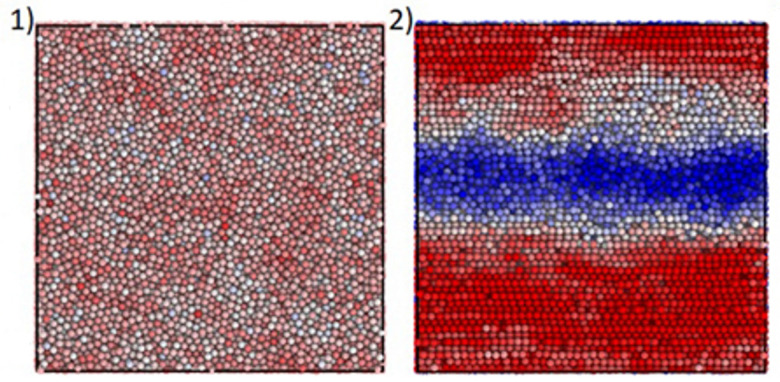
The shear-induced formation of a central low-concentrated layer (transition from the homogeneous suspension (**1**) to a stratified structure (**2**) Different colors correspond to various concentrations—blue 48%, red 58% [47] (with permission).

Figure 9 shows the stratification of the flow with a rather clear shear-induced separation of the central layer with a lower concentration in comparison with the higher concentration near the wall. Naturally, this stratification is associated with a decrease in “apparent” viscosity.

Can the concentration stratification of suspensions in flow be considered as the deformation-induced “phase separation” that happens in polymer solutions [79,80]? Situations presented in Figure 8 and Figure 9 cannot be considered as “phase” separation. However, it is this separation into highly concentrated composition with $\varphi ≅\varphi_{m}$ that possibly corresponds to mechanical glass transition; a low concentrated composition with $\varphi <\varphi_{m}$ corresponds to the flow state of suspensions. These effects are similar to shear banding, well known for polymer solutions [81,82,83,84]; macromolecular-like and worm-like micelles [85,86,87]; and, in general, for any complex liquids [88]. Similar shear banding was observed for colloidal dispersions [89,90] including an instability characteristic effect for such complex liquids [91].

**Figure 10:**
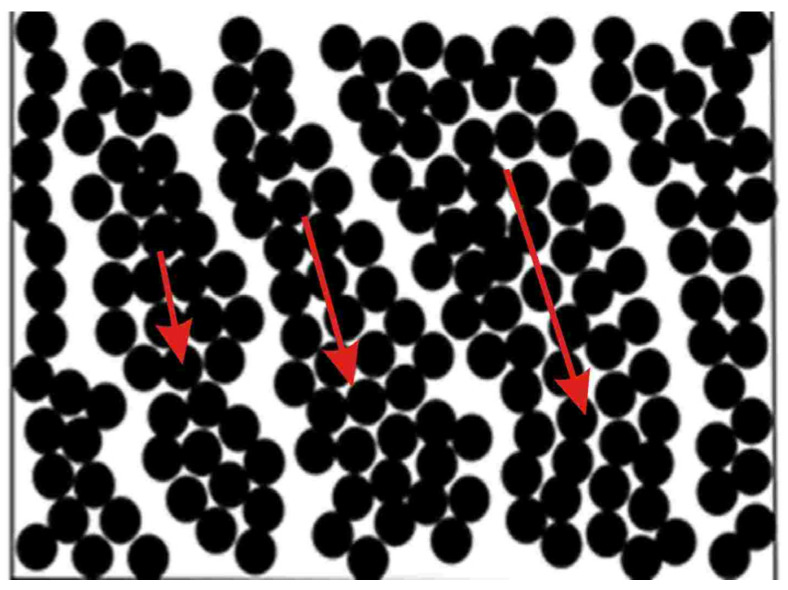
Slip of clusters in highly concentrated suspensions. Red arrows show the direction if shift.

For concentrated suspensions, the effect of heterogeneity is more specific, consisting of the disruption of flow continuity and the movement of individual blocks relative to each other across the interfaces. At high enough stresses, macro-cracks appear in concentrated suspensions dividing clusters, and they slip at their boundaries providing displacement of materials [92,93,94,95], as shown in Figure 10. It may look like flow, but this is not flow!

Decomposition in bulk and displacement of clusters is one of the possible reactions of concentrated suspensions to the impact of high stresses. If the attractive interaction between the particles is strong enough, the concentrated suspension resembles a stone and its reaction is to slide along the boundary surface [95]. A real or apparent slip in shear experiments with concentrated suspensions is quite possible [96,97,98].

**Figure 11:**
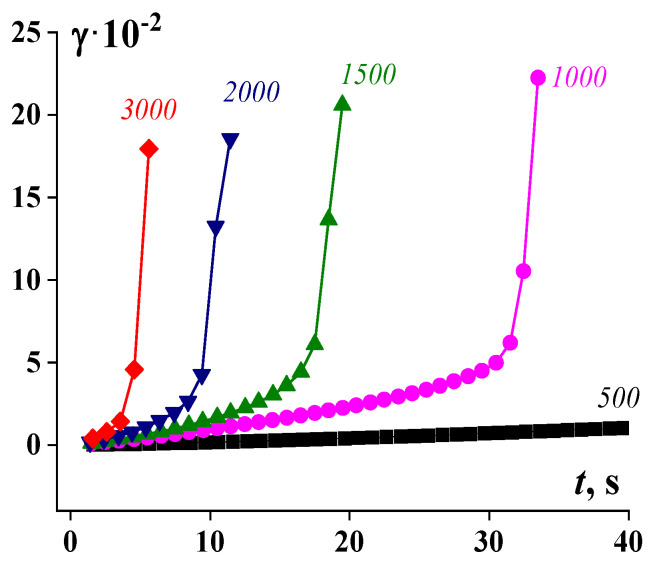
Development of deformation over time for 55% suspension at different given shear stresses (torques) measured on a rotational rheometer. Stresses (in Pa) are shown at the curves [99].

The picture illustrating the development of deformations in time with the increase in given shear stress (calculated from the measured torque) is shown in Figure 11. The spurt (sudden cutting increase in deformation) corresponds not to the fall of the apparent viscosity but to the detachment of the sample from the solid boundary surface (loss of the adhesive contact) and the occurrence of the slip.

**Figure 12:**
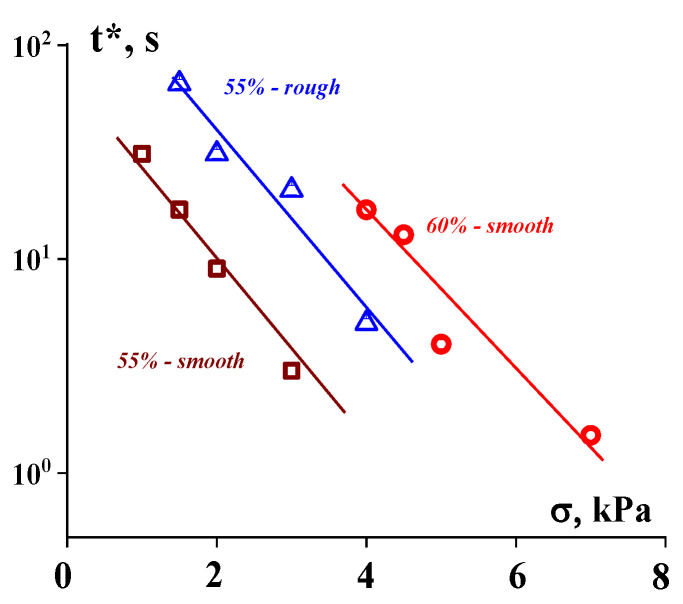
Durability of the adhesion strength of the suspension/surface contact. Concentration of the suspensions and the type of surface are shown at the curves [99].

The strength of the suspension/surface contact is characterized by a certain durability (lifetime), t*, at which a jump in shear rate is observed. This point depends on the stress that is typical for different materials (Figure 12).

The $t^{*}\left(\sigma \right)$ dependence is approximated using the usual Bueche–Zhurkov exponential function:

(6) $$t^{*}∼e^{-\alpha \sigma }$$

The constant α depends on the nature of the contacting materials and the geometry of the surface.

**Figure 13:**
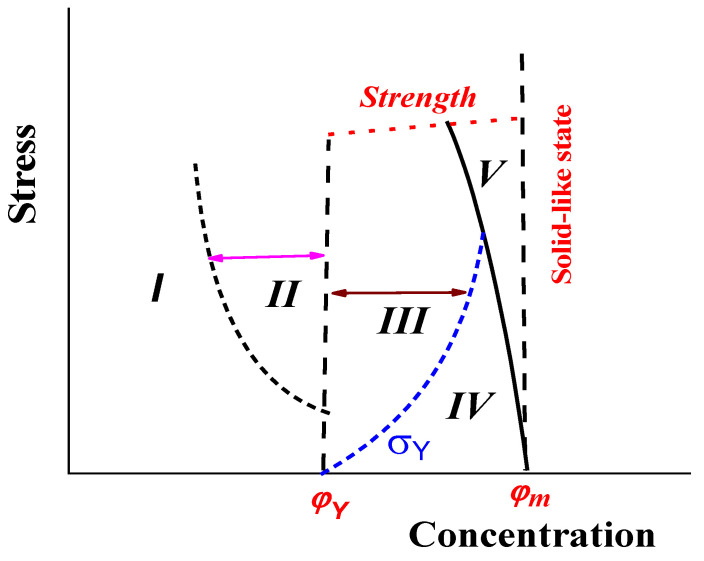
Diagram of the rheological states of suspensions dependent on concentration and applied stress. Comments—in the text.

The features of the rheological properties of concentrated suspensions discussed above provide the basis for their presentation in the form of a diagram shown in Figure 13. Region ***I*** in this diagram corresponds to dilute suspensions and low shear stresses of moderately concentrated suspensions. Non-linear effects (such as non-Newtonian flow and normal stresses in shearing) can be detected in sub-region ***II*** for moderately concentrated suspensions due to particle collisions, formation, and decomposition of temporary aggregates and distortions of flow lines. A permanent network in regions ***III***–***IV*** is responsible for the appearance of the yield stress and its lower boundary is marked as the threshold concentration *φ_Y_*. This is the area of yielding material (liquid) and the curve *φ_Y_* divides this area into the regions of liquid state (***III***) and gel-like state (***IV***) [2]. Finally, the narrow band ***V*** is the region of elasto-plastic behavior shown in Figure 13. The boundary line *φ_m_* is the limit of the possibly of filling and, at this concentration, the movement shown in Figure 10 is only possible. As discussed above, the boundaries of the regions corresponding to the different rheological behavior of suspensions depend on various factor and are due to the thixotropic effects on the time factor first of all.

Regions ***III***–***V*** are objects of powder injection molding technology, and one of the primary tasks in real practice is to shift the line *φ_m_* towards as high a value as possible and to compress region ***V***.

## 5. Conclusions and Perspectives

Highly concentrated filled polymer compositions belong to a class of suspensions—materials characterized as having specific rheological properties. These properties are determined via the packing mode and approaching the random or regular limit of volume filling degree, *φ_m_*. The limiting degree of random filling depends on the particle size, especially at the nano/microscale. An increase in the volume concentration can be achieved by using polydisperse particles. Compositions may flow at concentrations below this limiting degree of filling. At higher concentrations, small irreversible plastic deformations (but not steady flow) are possible, and further loading leads to jamming (formation of a plug), which is equivalent to the transition to a solid-like state. At concentrations close to the limiting *φ_m_*, an inhomogeneous structure appears in the form of self-organized clusters. These clusters are moving as a whole and contribute to the formation of a layered flow and instability of movement with the occurrence of huge oscillations in stresses and concentrations. Finally, when the strength limit of contacts between them is exceeded, macro-fractures appear. All these features of the rheological properties and structure of highly concentrated compositions play a key role in processing filled polymers and powder injection molding and require a reasonable choice of the content of composition, total concentration, and processing conditions which determine the structure and properties of the final product [100].

In addition to these general considerations, those areas of modern applications, where the use of highly filled polymer compositions is especially important, should be noted. These are additive technologies, as is the use of these composites in regenerative surgery. In the latter case, the physicochemical aspects of the behavior of filled polymers are especially important.

Of course, there are a number of problems beyond the scope of this review that will require further research. This applies to the following problems:

- Detailed consideration of the role of interparticle interaction, i.e., attraction or repulsion of filler particles;
- Interaction of the filler with the polymeric matrix;
- Assessment of the role of the shape of filler particles, i.e., transition from spherical to oblong particles;
- Dynamics of movement of filled compositions through channels of different geometry with formations of the surface layer and shear banding;
- Microfluidics, i.e., movement of filled liquids through channels, the size of which is commensurate with the size of the particles;
- Physics of plasticity in deformation of highly filled polymer compositions;
- Consideration of the rheology of polymer compositions with deformable particles, including foams.

## Figures and Tables

**Table 1:** Maximum possible particle concentrations in dense suspensions depending on the size of dispersed particles [24].

Qualification	Size of Particles	Maximum Possible Particle Concentrations, *φ_m_*
Nanoparticles	1–100 nm	0.05–0.20
Ultradispersed particles	0.1–1.0 μm	0.20–0.255
Microparticles	1.0–10 μm	0.255–0.45
Macroparticles	10–40 μm	0.45–0.62
Large particles	>50 μm	0.62–0.64

## Data Availability

Not applicable.
